# Establishing a continuum of acute kidney injury – tracing AKI using data source linkage and long-term follow-up: Workgroup Statements from the 15th ADQI Consensus Conference

**DOI:** 10.1186/s40697-016-0102-0

**Published:** 2016-02-26

**Authors:** Ravindra Mehta, Azra Bihorac, Nicholas M. Selby, Hude Quan, Stuart L. Goldstein, John A. Kellum, Claudio Ronco, Sean M. Bagshaw, For the Acute Dialysis Quality Initiative (ADQI) Consensus Group

**Affiliations:** Division of Nephrology and Hypertension, Department of Medicine, University of California, San Diego, CA USA; Department of Anesthesiology, University of Florida, Box 100254, Gainesville, FL 32610-0254 USA; Division of Health Sciences and Graduate Entry Medicine, University of Nottingham, Nottingham, UK; Department of Community Health Sciences, University of Calgary, Calgary, Canada; Division of Pediatric Nephrology, Department of Pediatrics, Cincinnati Children’s Hospital Medical Center, Cincinnati, OH USA; Department of Critical Care Medicine, Center for Critical Care Nephrology, CRISMA Centre, University of Pittsburgh School of Medicine, Pittsburgh, PA USA; Department of Nephrology Dialysis & Transplantation, San Bortolo Hospital and the International Renal Research Institute (IRRIV), 36100 Vicenza, Italy; Division of Critical Care Medicine, Faculty of Medicine and Dentistry, University of Alberta, Edmonton, Canada

**Keywords:** Acute kidney injury, Chronic kidney disease, Electronic health records, Interoperability, Longitudinal follow-up, Minimal data set, Big Data, Blue Button Initiative

## Abstract

**Background:**

Acute kidney injury (AKI) is independently associated with the development of chronic kidney disease, endstage kidney disease and increased all-cause and cardiovascular-specific mortality. The severity of the renal insult and the development of multiple AKI episodes increase the risk of occurrence of these outcomes. Despite these long-term effects, only a minority of patients receive nephrologist follow up after an episode of AKI; those that do may have improved outcomes. Furthermore, relatively simple quality improvement strategies have the potential to change this status quo.

**Methods:**

On this background, a working group of the 15^th^ Acute Dialysis Quality Initiative (ADQI) conference applied the consensus-building process informed by review of English language articles identified through PubMed search to address questions related to the opportunities, methodological requirements and barriers for longitudinal follow-up of patients with AKI in the era of electronic health records and Big Data.

**Results:**

Four consensus statements answering the key questions identified by the working group are developed.

**Conclusions:**

We have identified minimal data elements and potential data sources necessary to trace the natural history of patients from onset of AKI to long-term outcome. Minimum infrastructure and key barriers to achieving these goals are outlined together with proposed solutions.

## Abrégé

**Contexte motivant la revue:**

L’insuffisance rénale aigüe (IRA) est associée de manière indépendante à l’évolution de l’état de santé du patient vers l’insuffisance rénale chronique (IRC), l’insuffisance rénale terminale (IRT) et à l’augmentation de la mortalité due à des complications, incluant des événements cardiovasculaires. Le risque d’une aggravation de la maladie vers ces événements indésirables est augmenté par la sévérité et par la récurrence des épisodes d’IRA. Malgré cette possibilité d’évolution défavorable à long terme, seulement une minorité de patients bénéficie d’un suivi par un néphrologue à la suite d’un épisode d’IRA, et dans le cas de ces patients, un meilleur pronostic pourrait être envisagé. Qui plus est, de simples stratégies d’amélioration dans la qualité du suivi pourraient changer la situation.

**Méthodologie et sources de l’information:**

Lors du 15e Congrès de l’*Acute Dialysis Quality Initiative* (ADQI), un groupe de travail a mis en application un processus de concertation à la suite du passage en revue d’articles publiés en anglais sur PubMed. Les paramètres de recherche visaient à répondre à des questions concernant le bien-fondé, les exigences méthodologiques et les obstacles associés à un suivi longitudinal des patients souffrant d’IRA en cette ère de dossiers médicaux électroniques et de mégadonnées.

**Résultats:**

Le groupe de travail a rédigé quatre déclarations consensuelles qui répondaient aux paramètres de recherche mentionnés, elles sont discutées dans la présente revue.

**Limites de l’étude:**

Les auteurs n’ont pas procédé à une revue formelle et systématique de la littérature sur le sujet.

**Conclusions:**

Cette revue a rendu possible l’identification de données de base et de sources potentielles de données qui permettraient de suivre l’évolution naturelle de la maladie chez les patients, des premiers épisodes d’IRA jusqu’aux possibles complications ou événements indésirables à long terme. Les infrastructures minimales requises et les principaux obstacles à l’atteinte de cet objectif sont exposés conjointement avec les solutions proposées.

## Background

There is increasing concern as to the long-term consequences of patients with acute kidney injury (AKI). Adverse outcomes reported in survivors of AKI include an increased risk of Cchronic kidney disease (CKD) onset and progression and death from cardiovascular disease and from all causes [1–12]. Experimental data show similarities between the pathological processes that drive CKD progression and those that occur following an episode of AKI [13, 14]. AKI and CKD may best be viewed as inter-related syndromes with AKI leading to CKD and CKD strongly predisposing to the development of AKI [15]. AKI has also been linked to increased long-term risk of cardiovascular diseases [8, 16–19], stroke [20], infection [21] and fracture [22]. Despite these long-term effects, only a minority of patients receive follow up by a nephrologist after an episode of AKI; observational studies suggest that those that do may have improved outcomes compared with those who do not [23, 24]. Relatively simple quality improvement strategies have the potential to change this status quo [25]. On this background, a working group of the 15^th^ Acute Dialysis Quality Initiative (ADQI) conference sought to address the following four questions:

- Question 1: What would be the essential components necessary to track patients following an episode of AKI?
- Question 2: What data elements and sources would be ideally needed to trace the natural history of patients from onset of AKI to long-term outcome?
- Question 3: What would be the minimum infrastructure needs and support to enable data acquisition, compilation, storage, analysis and display for each purpose?
- Question 4: What are the key barriers to integrating these data sources across a common syndrome and tracking for longer term complications?

## Methods

The 15^th^ ADQI Consensus Conference Chairs convened a diverse panel representing relevant disciplines (i.e., nephrology, critical care, pediatrics, pharmacy, epidemiology, health services research, biostatistics, bioinformatics and data analytics) from five countries from North America and Europe around the theme of “Acute Kidney Injury in the Era of Big Data” for a 2-day consensus conference in Banff, Canada on September 6–8, 2015. This consensus meeting followed the established ADQI process, as previously described [26]. The broad objective of ADQI is to provide expert-based statements and interpretation of current knowledge for use by clinicians according to professional judgment and identify evidence-care gaps to establish research priorities. From this group, our work group was asked to examine the longitudinal follow up of AKI in the era of Big Data. We applied the consensus-building process informed by review of English language articles identified through PubMed search by working group members. We used search terms acute kidney injury, longitudinal data, long-term follow up, big data and data integration. We did not use a formal systematic review process. Group members performed an objective scientific review of the identified literature, developing a consensus of opinion, with evidence where possible, to distil current literature and articulate a research agenda to address important unanswered questions. A glossary of terms used is included in Table 1.

**Table 1:** Glossary of terms

Variable	Definition	Comments
Electronic Health Record (EHR)	The Electronic Health Record is a longitudinal electronic record of patient health information generated by one or more encounters in any care delivery setting. Included in this information are patient demographics, progress notes, problems, medications, vital signs, past medical history, immunizations, laboratory data and radiology reports. The EHR has the ability to generate a complete record of a clinical patient encounter, as well as supporting other care-related activities directly or indirectly via interface, including evidence-based decision support, quality management and outcomes reporting [31].	EHR serve as one source of data from which pertinent health information can be obtained. However, EHR are not ubiquitous and may result in incomplete data particularly as patients transition from one location to another.
Acute Kidney Injury (AKI)	Evidence of an acute decline in kidney function characterized by an elevation in serum creatinine or reduction in urine output over a short interval of 48 h to 7 days [36]. Requires documentation of a measurable change in renal function in relation to a reference point.	The time of diagnosis, maximum stage reached, clinical features (oliguric or not) and should be recorded. If ancillary criteria are used e.g. decline in creatinine these should be identified.
AKI Episode	A discrete time period recognized with a starting point when diagnostic criteria are present and ending when there is evidence of improvement in renal function to meet criteria of recovery. Repeat episodes should be identified by evidence of a new decline in renal function following an improvement in renal function	It is often difficult to determine exact start and end points of an AKI episode when creatinine and urine output are fluctuating. Criteria for classifying discrete episodes of AKI need to be developed.
AKI Follow-up	Assessment of clinical and lab data at specific intervals following an AKI episode. Follow up should determine the level of general heath, level of renal function, consequences of the AKI on target organs and functional status and assess modifiable factors influencing outcomes. Specific interventions to improve recovery should be considered at each follow up visit.	Data recording should distinguish single from multiple episodes that may be contiguous or separated in time. Analysis of the trajectory of serum creatinine changes could be used to track individual episodes. An electronic “tag” should be placed in patients record identifying the index episode to enable them being recognized as high risk for subsequent events.
Minimal Data set	Set of variables that specifies the common data elements (CDE) that would be extracted at different time points after an episode of AKI (and then could be supplemented by additional data items).	Core data elements should include: when the AKI episode occurred, location, etiology, associated events, key features in management, course, consequences and outcomes). The frequency of recording would be defined by best practices to allow timely interventions at patient centric levels. This minimum dataset would be the basis for both patient centric and population level tracking across geographic areas or jurisdictions.
Unique patient identifiers (UPI)	A system that assigns individuals a unique number (the healthcare version of a Social Security Number) as a tool for patient identification across the different health care systems.	Not available in all countries and settings.
Blue Button Initiative	A system allowing patients and consumers access to their health records electronically through the “Blue Button” *mechanism* which allows consumers to take download and use their own health information.	Blue Button originated at the Veteran’s Administration as a symbol on its patient portal that beneficiaries could click to securely download their own health record electronically. Since then the Blue Button has spread beyond VA to other to more than 450 government and the private sector organizations making personal health data available to Americans.
SNOMED	The Systematized Nomenclature of Medicine is a systematic, collection of medical terms amenable for computer processing. It provides codes, terms, synonyms and definitions which cover anatomy, diseases, findings, procedures, microorganisms, substances, etc.	
Interoperability	The ability of a system to exchange electronic health information with and use electronic health information from other systems without special effort on the part of the user. Interoperability is made possible by the implementation of standards.	http://www.ieee.org/publications_standards/index.html
Renal functional recovery from AKI	Evidence of improvement in renal function to a level close to the reference point. There are variable definitions of complete and partial recovery in different studies [59, 60]	Determining recovery is often difficult as there may be inadequate follow up of clinical and lab data as patients may be seen in different locations under different providers and systems. This is much easier when patients are cared for in a single health care system with shared data (e.g Veterans Affairs medical centres in the US or UK NHS)
Mortality	Documentation of death, cause, contributing factors and time to death from onset of AKI.	This endpoint should be measured at several time points from AKI diagnosis but at a minimum at hospital discharge and at 90 days post AKI.
Chronic kidney Disease (CKD) Status	State of kidney health prior to development of AKI based on historical data.	Consistency in determining and recording CKD stage is necessary. We recommend using consensus staging criteria and validated equation to calculate estimated glomerular filtration rate.

## Results

### Question 1: What would be the essential components necessary to track patients following an episode of AKI?

#### Consensus statement 1A

- Short and long-term outcomes following an episode of AKI should be tracked at both patient and population levels to allow appropriate patient follow up and management while concurrently enabling data capture on the burden and outcomes of AKI.

#### Consensus statement 1B

- Patient level data needs to be sufficiently granular to inform clinical diagnosis, management and processes of care. Population level data may be collected periodically from different sources and should link all AKI episodes to relevant kidney health and overall long-term outcomes.

Tracking the outcomes of patients who have sustained AKI should be a powerful mechanism in improving standards of care, with the potential to effect change through a variety of means. The process can be considered at individual patient or at population levels; a patient-centred approach must take account of the fact that nephrologist follow up after an episode of AKI often does not occur in current practice [23]. In part, this may reflect a lack of awareness amongst patients that they had developed AKI, and among providers for the need for specific follow up, thereby constraining subsequent patient-driven health care encounters. The primary aim of improved patient-level tracking would be to educate patients on the importance of kidney health and to allow providers to deliver supportive renal care to patients in a more systematic way. Justification for this approach is provided by the expert onion of ADQI members and limited observational studies showing that focused follow up with a nephrologist may improve outcomes, by reducing the risk of recurrent AKI [24]. In tandem, improving identification and tracking of AKI episodes at a population level would advance the measurement of trends in the prevalence of AKI and its outcomes. Such approaches would move away from problems inherent with utilization of administrative codes for case finding (in particular a lack of sensitivity [27]) and aim to be inclusive of all AKI episodes, with subsequent enrichment by linkage to other data sources. This would allow descriptions of the burden of AKI across health care systems, and may lead to descriptions of the patient characteristics that do and do not benefit from specific follow up, or who are likely to benefit from emerging therapies. Opportunities for organisational-level quality improvement would also result (e.g., benchmarking) and this approach may even provide the basis for registry-based clinical trials [28]. Such outputs from population level data analysis could then feed back into patient level care by informing the refinement of care pathways and prognosis.

**Fig. 1:**
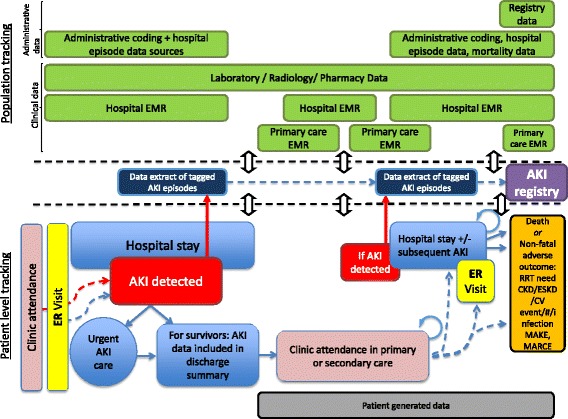
Diagram of data collection opportunities across the acute kidney injury patient pathway. Reproduced with permission from ADQI

An expanding literature showing the diversity of adverse outcomes associated with AKI means that the nature of outcomes to be measured requires specific consideration (Table 2). As well as renal outcomes (renal function, albuminuria and requirement for renal replacement therapy, RRT), systemic health consequences such as cardiovascular events and even increased rates of infection and fractures are relevant [8, 16, 18–22]. Furthermore symptom burden, quality of life and functional status are the least studied outcomes although arguably the most important from a patient’s point of view. Each will have different measurement requirements, varying sources from which data may be collected and require different timeframes for follow up (Fig. 1). For example, AKI may result in end stage kidney disease (ESKD) requiring dialysis at any time-point following its onset, whereas cardiovascular consequences are unlikely to occur until year(s) after.

**Table 2:** Possible outcome measures for use in tracking episodes of AKI

	Entity resolution	Initial encounter		Subsequent encounters	
		Kidney Health	Overall health	Kidney Health	Overall health
Population	Name Unique identifier Gender Date of birth	AKI detection results/tag. Serum creatinine measurements (baseline, during AKI and recovery).	ICD codes Comorbidities Complications Resource utilization e.g. length of stay, critical care admission.	All subsequent AKI episodes, stages and timing. eGFR. Albuminuria. CKD stage*.* Need for RRT.	Re-hospitalization. New diagnoses, ICD codes: CV events, cancer, infection, fracture, MAKE, MARCE. Time and cause of death.
Patient	Name Unique identifier Sex Date of birth	AKI detection results/tag AKI stage, duration and setting AKI etiology Need for acute RRT Nephrology consult	Setting of AKI, exposures and co-existing illness. Blood pressure. Procedures.	eGFR. Albuminuria. CKD stage. Need for RRT. Blood pressure.	Comorbidities. Complications. Quality of life/dependency.
Management	Name Unique identifier Gender Date of birth	Treatment given for AKI. AKI follow up plans. Medications. Discharge quality indicators. Education (including patient).	Medications (including those stopped/temporarily suspended). Frailty measures. Patient reported outcomes and symptoms.	CKD care. Secondary prevention of AKI episodes. Medications.	Functional recovery. Cardiovascular risk. Frailty measures. Patient reported outcomes and symptoms.
Research		Biomarkers.	Specific therapies. Improved methods of capturing patient reported data.	Therapies to facilitate recovery. Use of biomarkers to predict risk/recovery.	Therapies to improve systemic outcomes.

*AKI* acute kidney injury, *ICD* International Classification of Diseases, *eGFR* estimated glomerular filtration rate, *CKD* chronic kidney disease, *RRT* renal replacement therapy, *MAKE* major adverse kidney events, *MARCE* major adverse renal and cardiovascular events

On a patient level, the individual clinical situation will dictate how soon and how often a patient requires follow up after an AKI episode, but follow up and subsequent utilization of health care will ultimately be determined by patients’ choices and behaviour. This will dictate the frequency of data collection and even whether this happens at all. As well as targeting improved follow-up rates (e.g., by empowering patients to take interest in their kidney health), the precision and level of detail in clinical records is also important [29]. An accurate summary of the AKI episode in discharge documentation is a key element, transferring information to primary care and supporting better patient and provider awareness. Information pertaining to the when, where and why the episode of AKI occurred should be recorded, as well as how it was managed. Follow-up requirements should be specified (including clinic appointments, clinical decision support on timing of renal function checks and communicating medication plans) and this will also provide reference information for subsequent hospital based encounters. In the UK, this has been designated a priority, with commissioning policy shaped to target improvements in this area [30]. In time, improved granularity of patient-level data would not only aid clinical care but also serve to improve the quality of population-level data collection.

In the US, the American Recovery and Reinvestment Act and the Health Information Technology for Economic and Clinical Health Act have driven nationwide uptake and usage of electronic health records (EHR). Ideally, the EHR should provide the solution to tracking AKI outcomes, as per the Health Information Management Systems Society definition (Table 1) [31]. However, there are several factors that mean that this is not a current or complete solution. Firstly, many of EHR in current usage are fragmented, relying on a large number of different platforms (e.g., admission, discharge and transfer, computer-based provider order entry, clinical decision support system, laboratory information system (LIMS) and imaging archiving and communication system) so that patient data are not always linked [32]. A number of concerns have been raised around injudicious use of erroneous, miscoded, fragmented and incomplete data from EHR, recognizing that the quality of clinician-entered data can contribute to this [29]. The use of EHR is not ubiquitous, even in developed countries, thereby limiting the capture of certain types of data. Furthermore, the integration of EHR outside individual institutions or organizations does not commonly exist. It can also be a challenge to identify the pertinent data from within the wealth of data contained within the EHR. Data extracts must select the most relevant; one model would be to define a minimal dataset that specifies the common data elements (CDE) that would be extracted at different time points after an episode of AKI (and then could be supplemented by additional data items). This minimum dataset would be the basis for both patient-centred and population-level tracking across geographic areas or jurisdictions.

### Question 2: What data elements and sources would be ideally needed to trace the natural history of patients from onset of AKI to long-term outcome?

#### Consensus statement 2A

- Methodology to detect and define an episode of AKI must be standardised (and based on current diagnostic criteria) if data are to be objectively combined from multiple sources and locations.

#### Consensus statement 2B

- For both patient and population tracking the patient should be the unit of observation.

#### Consensus statement 2C

- Relevant clinical information pertaining to an episode of AKI should be included in a patient’s health care encounter (including occurrence and stage of AKI as well as a follow-up plan). As a minimum follow-up requirement, renal function and albuminuria should be checked within three months of an AKI encounter (as per KDIGO guidelines).

Despite the widespread acceptance of international consensus criteria for AKI, it is still possible to introduce considerable variation when applying these in clinical practice. A particular challenge is selection of an individual’s baseline or reference serum creatinine, with different methodologies producing varied diagnostic thresholds [33]. It can be particularly difficult in those patients with no previous serum creatinine results [34, 35]. Therefore, a core requirement is that a standardised approach is adopted for detecting AKI if data are to be combined from different sources and across health care locations. Failure to do so will result in invalid comparisons between groups of patients in whom AKI has been detected in different ways. Not only must the onset of AKI be defined, but also what constitutes an ‘episode’ of AKI (Table 1). However, even with a standardised detection method, collection of data that indicates the onset of AKI can occur at different levels, ranging from individual laboratory results indicating AKI, to episodes of AKI (both of which may occur singly or multiply for an individual) through to data collection on a patient level. As multiple episodes of AKI in an individual may exert cumulative effects [9], and because the majority of outcomes (such as mortality, CKD progression, cardiovascular events) are measured in an individual, the primary method of tracking outcomes should be at a patient level (i.e. the patient as the unit of observation). However, it is important to capture occurrence and timing of multiple AKI episodes as some analyses may need to be performed at this level (e.g., short term consequences of an AKI episode).

This methodology aligns with a concept of applying a ‘tag’ to patients’ medical records once AKI has been detected (incidence), which then triggers follow up at both patient and population levels (the location and nature of this tag may vary depending on local capability, but would have to be sufficiently apparent so it would be useful for clinical practice). Timing of the index AKI episode would be used as the reference for subsequent outcomes, including recurrent episodes of AKI. A minimum dataset should be captured for each patient to track post-AKI outcomes. The dataset would need to be able to be collected at different longitudinal time points, and identify both the patient and occurrence of subsequent events. At its most basic, this would include a population specific unique patient identifier(s), alongside an assessment of renal function (serum creatinine, eGFR, albuminuria or urinalysis, subsequent AKI detection results, need for RRT), each with a date and time stamp. In terms of minimum requirement for individual patient follow up, in the absence of evidence to the contrary we support the KDIGO recommendation that all patients who have sustained AKI should have renal function and albuminuria assessed within three months [36]. This also provides a process measure that may be appropriate as a clinical quality standard.

A more comprehensive dataset may include other systemic outcomes (physiological parameters such as blood pressure, or systemic consequences such as cardiovascular events) although an alternative approach would extract and retrospectively link this type of data from other sources, particularly those that are binary occurrences. Longitudinal prescribing information also would have value. Table 2 summarises potential outcomes measures at patient and population levels, stratified into potential ease or difficulty of collection at the present time. There are also some technical considerations including the need for methods to deal with the use of different units of measurement for serum creatinine (μmol/L versus mg/dL), so that conversion and merging of data are possible. Precision in defining data fields is also essential and the use of the standardized computer nomenclature (Systematized Nomenclature of Medicine (SNOMED)) to reduce inconsistency in information capture and to facilitate interchange of information across EHR is critical [37].

If a core dataset is then to be enriched by linkage, the other data sources that may be utilised are multiple and varied. As previously described, it is possible classify these into administrative data, clinical data and patient-collected data [38], see Table 3. This then allows a patient’s journey to be described in parallel with the locations and time points at which they may subsequently access healthcare and the multiple data sources that document encounters. These healthcare utilisation episodes therefore form opportunities from which outcome data can be measured. We have attempted to demonstrate this in Fig. 1; the patient remains at the centre of both individual and population level approaches.

**Table 3:** Types of data that could be utilised to track AKI (adapted from Deeny et al [38])

Data type	Definition	Characteristics	Examples
Administrative data	Data collected as part of the routine administration of healthcare, for example reimbursement and contracting.	Records of attendances, procedures and diagnoses entered manually into the administration system for a hospital or other healthcare organization and then collated at regional or national level. Little or no patient or clinician review; no data on severity of illness.	Hospital episode statistics (England): Clinical coders review patients’ notes, and assign and input codes following discharge. These codes are used within a grouper algorithm to calculate the payment owed to the care provider. Veterans administration databases: Data from health care episodes within the VA system for both in-patient and out-patient treatment.
Clinical data	Data collected by healthcare workers to provide diagnosis and treatment as part of clinical care. These data might arise from the patient (for example, reports of symptoms) but are recorded by the clinician.	Electronic medical record of patient diagnoses and treatment. Results of laboratory tests. Compared with administrative data, less standardized in terms of the codes used and less likely to be collated at regional and national levels.	Electronic medical record: More than 90 % of primary care doctors reported using the Electronic Medical Record (EHR) in Australia, the Netherlands, New Zealand, Norway and the UK in 2012. In the US, the American Recovery and Reinvestment Act and the Health Information Technology for Economic and Clinical Health Act have driven nationwide uptake and usage of electronic health records (EHR).
Patient generated data	Data requested by the clinician or healthcare system and reported directly by the patient to monitor patient health, as well as data that the individual decides to record autonomously without the direct involvement of a health care practitioner.	Data collected by the patient on clinical metrics (eg, blood pressure), symptoms or patient reported outcomes; also symptoms and treatment recorded by the patient outside the ‘traditional’ healthcare system structures.	Examples include telehealth (e.g. for heart failure patients), UK (Renal) PatientView that allows patients to upload blood pressure, weight and glucose measurements (https://www.patientview.org), Patients Like Me an online quantitative personal research platform (http://www.patientslikeme.com), and individual and patient activity on social media.
Machine generated data	Data automatically generated by a computer process, sensor etc. to monitor staff or patient behavior passively.	Record of individual behavior as generated by interaction with machines. The nature of the data recorded is determined by the technology used and substantial processing is typically required to interpret it	Telecare sensors: Telecare aims for remote, passive and automatic monitoring of behavior within the home, for example for frail older people.

### Question 3: What would be the minimum infrastructure needs and support to enable data acquisition, compilation, storage, analysis and display for each purpose?

#### Consensus statement 3A

- A first episode of AKI should add an AKI identifier (tag) to the patient record that would allow subsequent identification of the episode that would enable follow up. Inclusion of a population-based unique patient identifier within the patient record will facilitate database linkage and avoid duplication of patient medical records.

#### Consensus statement 3B

- Patients who have sustained an episode of AKI should be educated about the diagnosis and follow up arrangements discussed with them.
- Providers responsible for patient care should be informed of the patients AKI episode and need for follow up at specific intervals.

We consider the first episode of AKI as a sentinel event that initiates “tagging” of patient’s record with AKI diagnosis to prompt healthcare provider to initiate longitudinal follow-up. The process may be as simple as addition of appropriate diagnostic codes to problem list and discharge summary within EHR. Several observational studies have reported low prevalence of reported AKI diagnoses in discharge summaries [1, 39–41] even when AKI diagnosis was ascertained with electronic alerts. More comprehensive approach would require definition of common data elements (CDE) necessary to capture AKI episode. The development of CDE requires a standardized process for structured data capture, such as definition and capture of relevant data elements into templates that can be automatically populated from an EHR and a standard way to access, display and store the data. The CDE would act as a template that can be integrated in different EHR platforms and transmitted and shared among healthcare providers and organizations.

The Office of the National Coordinator for Health Information Technology (ONC), in partnership with National Library of Medicine and Agency for Healthcare Research and Quality has already recognized importance of structured data capture for patient-centered integration of the EHR into other parts of the learning health care system such as research consortia, registries and public health agencies. Among others, the Standards and Interoperability Framework Initiative [42] has resulted in development of CDAs for patient-centered outcomes reporting [43] and the common formats for safety reporting [44]. The interest of multiple stakeholders involved in these initiatives needs to be engaged in understanding the importance of defining common data elements for both acute and chronic kidney disease from both patient and public health perspective. Engagement of representatives from professional societies such as American Society of Nephrology, International Society of Nephrology, National Kidney Foundation and Society of Critical Care Medicine, is necessary to ensure uniform and standards. The recent position paper by National Kidney Disease Education Program outlines use of EHR for tracking in of CKD and provides excellent framework for expanding the issue to AKI patients [45].

In United States, the lack of unique patient identifiers (UPI) and current state of health information technology (IT) with inadequate EHR interoperability between different hospital systems, outpatient healthcare providers and laboratory services impose major obstacle for longitudinal follow-up after the initial AKI episode. The idea behind UPI is that system would allow integration of care delivery for individual patient across entire health care delivery system. The lack of such system impose major barriers in tracking disease progression for conditions for which awareness in society, among patients and healthcare providers is low, such as kidney disease and sepsis. Both AKI and sepsis demonstrate recidivism, can occur over time in different settings (hospital or community) and their recurrence can lead to development of chronic conditions affecting different organs [7, 9, 10]. The implementation of a national UPI system that assigns individuals a unique number (the healthcare version of a Social Security Number) has been debated as a tool for patient identification across the different health care systems in United States. In United Kingdom, Canada, Australia and New Zealand such systems are already in place. In United Kingdom the NHS number has served this purpose since 2009 [46]. Healthcare identifiers, unique 16 digit numbers assigned to individual patient, healthcare provider and healthcare organizations, are the building block for eHealth in Australia [47].

The need for the improvement in existing health IT infrastructure to support EHR interoperability is identified as the key strategic goal in the recently released Federal Health IT Strategic Plan 2015-2020 [48]. The advancement of person-centered health and wellness through the use of technology and health information is set as the main strategic national goal. The key element of the plan is development of the roadmap for EHR interoperability and advancement of technical standards to assure secure and interoperable health IT. Enabling structured data capture within EHR is poised to be a critical way to integrate EHR data into a variety of health services and clinical research activities and will constitute the important aspect of health IT development in the next 5 years that can be used as leverage for development of standardized longitudinal follow-up for AKI.

### Question 4: What are the key barriers to integrating these data sources across a common syndrome and tracking for longer term complications?

#### Consensus statement 4

- Multiple barriers to integrating data sources currently exist at patient, organizational and national levels.
- Strategies to tackle these barriers need to be developed and should include improvements in linkage between currently fragmented health record systems, and facilitating patient ownership of relevant medical information.

Patients who develop AKI are often cared for by different providers during an episode than those who will follow them subsequently, resulting in fragmentation of care. This is further compounded by differences in the nature and frequency of data that is recorded in the inpatient and outpatient setting. For instance while an EHR may be available in a hospital, when the patient is transferred to a skilled nursing facility only paper records maybe utilized and the lab studies would not be available. Strategies to scan and upload paper records into EHR are one possible solution to tackle this problem. It is thus imperative that efforts to integrate data across systems be encouraged to permit data sharing. In Southern California an initiative to share patient data across different health care systems is being tested [49].

A second barrier is educating patients and equipping them with tools to manage their health. The health care delivery systems account for only about 10–20 % of health outcomes. Most individuals are more often passive recipients of health care and long-term services and supports rather than informed, active partners who collaboratively make decisions [50]. Health care providers and health insurers offered fewer than three in ten individuals electronic or online access to their medical record in 2013 [51]. Progression to a patient-centered health IT infrastructure is recognized as another strategic goal for the next decade with emphasis on the use of novel tools. The Blue Button Initiative is one of the tools developed to enable individuals to securely access, manage and control their electronic health information [52] and would be an important tool to allow patients to have access to their tagged AKI information. The use of telehealth, virtual medicine and innovative technologies such as sensors and mobile technology could be a critical resource to offset the cost and improve compliance and easiness for longitudinal follow up after AKI.

Thus the informed patient plays a central role in the longitudinal follow-up after initial AKI episode. The development and dissemination of the tools and educational resources related to AKI to help patients understand their health information, costs, and care options and become advocates for their own health is critical and needs to occur at the time of hospital discharge.

An additional area that needs to be addressed is provider education and ensuring handoffs to track follow ups. Fewer than 50 % of patients with the most severe AKI will have a follow-up creatinine measured within the first three months of hospitalization, and among AKI survivors with persistent renal dysfunction at discharge, the referral rates for outpatient nephrology consultation are as low as 11 % [23, 53]. Since documentation of AKI episode is lacking in discharge summaries for more than half of the patients and follow up occurs for even fewer patients, it is plausible to assume that majority of patients with AKI may never become aware of the diagnosis and associated risk for adverse long-term outcomes [1, 39, 54, 55]. We propose to set of core required information needs to be included at the time of discharge and that could eventually become core measures that can be tracked across organizations and linked to quality of care.

Patients with greater clinical need (and therefore potentially at higher risk of adverse outcomes) are more likely to be higher users of heath care. This may risk ascertainment or selection bias [56–58] that can affect population measurement for some outcomes (e.g. assessment of renal function) more than others (e.g. myocardial infarction, or mortality).

### Research questions

- What mechanisms could be utilised to track patients lost to follow up: e.g., could an AKI tag be recorded in a central registry similar to a CDC surveillance of TB or HIV cases?
- Can a minimal data set be used for AKI surveillance in populations and for development and surveillance of quality measures?
- Can methods be developed to harmonize data between patient and population levels, including the use of data from multiple different sources?
- What approaches or specific therapies are effective in post AKI care?
- How can methods be developed to stratify patients into low and high risk of adverse consequences following AKI; how can this lead to the development of strategies for individualizing medical care?

## Conclusions

The ADQI authors agreed that outcomes following an episode of AKI should be tracked at both patient and population levels to allow appropriate patient follow up and management while concurrently enabling data capture on the burden and outcomes of AKI. Methodology to detect and define an episode of AKI must be standardized using current diagnostic criteria in order to effectively combine data from multiple sources and locations. For both patient and population tracking the patient should be the unit of observation. Relevant clinical information pertaining to an episode of AKI should be included in a patient’s health care encounter (including occurrence and stage of AKI as well as a follow-up plan). As a minimum follow-up requirement, renal function and albuminuria should be checked within three months of an AKI encounter. Currently multiple barriers for data integration exist at patient, organizational and national levels. Strategies to tackle these barriers need to be developed and should include improvements in linkage between currently fragmented health record systems, and facilitating patient ownership of relevant medical information.

## References

[1] Bihorac A, Yavas S, Subbiah S, Hobson CE, Schold JD, Gabrielli A (2009). Long-term risk of mortality and acute kidney injury during hospitalization after major surgery. Ann Surg.

[2] Hobson CE, Yavas S, Segal MS, Schold JD, Tribble CG, Layon AJ (2009). Acute kidney injury is associated with increased long-term mortality after cardiothoracic surgery. Circulation.

[3] Wald R, Quinn RR, Luo J (2009). Chronic dialysis and death among survivors of acute kidney injury requiring dialysis. JAMA.

[4] Amdur RL, Chawla LS, Amodeo S, Kimmel PL, Palant CE (2009). Outcomes following diagnosis of acute renal failure in U.S. veterans: focus on acute tubular necrosis. Kidney Int.

[5] Lo LJ, Go AS, Chertow GM, McCulloch CE, Fan D, Ordonez JD (2009). Dialysis-requiring acute renal failure increases the risk of progressive chronic kidney disease. Kidney Int.

[6] van Kuijk JP, Flu WJ, Chonchol M, Hoeks SE, Winkel TA, Verhagen HJ (2010). Temporary perioperative decline of renal function is an independent predictor for chronic kidney disease. Clin J Am Soc Nephrol.

[7] Ishani A, Nelson D, Clothier B, Schult T, Nugent S, Greer N (2011). The magnitude of acute serum creatinine increase after cardiac surgery and the risk of chronic kidney disease, progression of kidney disease, and death. Arch Intern Med.

[8] James MT, Ghali WA, Knudtson ML, Ravani P, Tonelli M, Faris P (2011). Associations between acute kidney injury and cardiovascular and renal outcomes after coronary angiography. Circulation.

[9] Thakar CV, Christianson A, Himmelfarb J, Leonard AC (2011). Acute kidney injury episodes and chronic kidney disease risk in diabetes mellitus. Clin J Am Soc Nephrol.

[10] Coca SG, Singanamala S, Parikh CR (2012). Chronic kidney disease after acute kidney injury: a systematic review and meta-analysis. Kidney Int.

[11] Chawla LS, Amdur RL, Amodeo S, Kimmel PL, Palant CE (2011). The severity of acute kidney injury predicts progression to chronic kidney disease. Kidney Int.

[12] Ozrazgat-Baslanti T, Thottakkara P, Huber M, Berg K, Gravenstein N, Tighe P, et al. Acute and chronic kidney disease and cardiovascular mortality after major surgery. Ann Surg. 2015. 10.1097/SLA.0000000000001582 PMC4936961 26756753

[13] Chawla LS, Kimmel PL (2012). Acute kidney injury and chronic kidney disease: an integrated clinical syndrome. Kidney Int.

[14] Venkatachalam MA, Griffin KA, Lan R, Geng H, Saikumar P, Bidani AK (2010). Acute kidney injury: a springboard for progression in chronic kidney disease. Am J Physiol Renal Physiol.

[15] Chawla LS, Eggers PW, Star RA, Kimmel PL (2014). Acute kidney injury and chronic kidney disease as interconnected syndromes. N Engl J Med.

[16] Chawla LS, Amdur RL, Shaw AD, Faselis C, Palant CE, Kimmel PL (2014). Association between AKI and long-term renal and cardiovascular outcomes in United States veterans. Clin J Am Soc Nephrol.

[17] Olsson D, Sartipy U, Braunschweig F, Holzmann MJ (2013). Acute kidney injury following coronary artery bypass surgery and long-term risk of heart failure. Circ Heart Fail.

[18] Liotta M, Olsson D, Sartipy U, Holzmann MJ. Minimal changes in postoperative creatinine values and early and late mortality and cardiovascular events after coronary artery bypass grafting. Am J Cardiol. 2014;113(1):70-5. doi: 10.1016/j.amjcard.2013.09.012. 10.1016/j.amjcard.2013.09.012 24176074

[19] Wu VC, Wu CH, Huang TM, Wang CY, Lai CF, Shiao CC (2014). Long-term risk of coronary events after AKI. J Am Soc Nephrol.

[20] Wu VC, Wu PC, Wu CH, Huang TM, Chang CH, Tsai PR, et al. The impact of acute kidney injury on the long-term risk of stroke. J Am Heart Assoc. 2014;3(4). doi:10.1161/jaha.114.000933. 10.1161/JAHA.114.000933 PMC4310379 25027018

[21] Lai TS, Wang CY, Pan SC, Huang TM, Lin MC, Lai CF (2013). Risk of developing severe sepsis after acute kidney injury: a population-based cohort study. Crit Care.

[22] Wang WJ, Chao CT, Huang YC, Wang CY, Chang CH, Huang TM (2014). The impact of acute kidney injury with temporary dialysis on the risk of fracture. J Bone Miner Res.

[23] Siew ED, Peterson JF, Eden SK, Hung AM, Speroff T, Ikizler TA (2012). Outpatient nephrology referral rates after acute kidney injury. J Am Soc Nephrol.

[24] Xie M, Iqbal S (2014). Predictors for nephrology outpatient care and recurrence of acute kidney injury (AKI) after an in-hospital AKI episode. Hemodial Int.

[25] Silver SA, Harel Z, Harvey A, Adhikari NK, Slack A, Acedillo R (2015). Improving Care after Acute Kidney Injury: A Prospective Time Series Study. Nephron.

[26] Kellum JA, Bellomo R, Ronco C (2008). Acute Dialysis Quality Initiative (ADQI): methodology. Int J Artif Organs.

[27] Tomlinson L, Riding A, Payne R, Abel G, Tomson C, Wilkinson I (2013). The accuracy of diagnostic coding for acute kidney injury in England - a single centre study. BMC Nephrol.

[28] Selby NM, Hill R, Fluck RJ, Programme NHSETKA. Standardizing the Early Identification of Acute Kidney Injury: The NHS England National Patient Safety Alert. Nephron. 2015. doi:10.1159/000439146. 10.1159/000439146 26351847

[29] Hoffman S, Podgurski A (2013). Big Bad Data: Law, Public Health, and Biomedical Databases. J Law Med Ethics.

[30] NHS England. Commissioning for Quality and Innovation (CQUIN) Guidance for 2015/16. https://www.england.nhs.uk/wp-content/uploads/2015/03/9-cquin-guid-2015-16.pdf.

[31] Healthcare Information and Management Systems Society (HIMSS). Electronic Health Records. 2015. http://www.himss.org/library/ehr/.

[32] Kashani K, Herasevich V (2015). Utilities of electronic medical records to improve quality of care for acute kidney injury: past, present, future. Nephron.

[33] Lafrance JP, Miller DR (2010). Defining acute kidney injury in database studies: the effects of varying the baseline kidney function assessment period and considering CKD status. Am J Kidney Dis.

[34] Bagshaw SM, Uchino S, Cruz D, Bellomo R, Morimatsu H, Morgera S (2009). A comparison of observed versus estimated baseline creatinine for determination of RIFLE class in patients with acute kidney injury. Nephrol Dial Transplant.

[35] Siew ED, Matheny ME, Ikizler TA, Lewis JB, Miller RA, Waitman LR (2010). Commonly used surrogates for baseline renal function affect the classification and prognosis of acute kidney injury. Kidney Int.

[36] KDIGO AKI Work Group (2012). Clinical Practice Guideline for Acute Kidney Injury. Kidney Int Suppl.

[37] Ruch P, Gobeill J, Lovis C, Geissbuhler A. Automatic medical encoding with SNOMED categories. BMC Med Inform Decis Mak. 2008;8(Suppl 1):S6. doi:10.1186/1472-6947-8-S1-S6. 10.1186/1472-6947-8-S1-S6 PMC2582793 19007443

[38] Deeny SR, Steventon A (2015). Making sense of the shadows: priorities for creating a learning healthcare system based on routinely collected data. BMJ Qual Saf.

[39] Vaught AJ, Ozrazgat-Baslanti T, Javed A, Morgan L, Hobson CE, Bihorac A (2015). Acute kidney injury in major gynaecological surgery: an observational study. BJOG.

[40] Huber M, Ozrazgat-Baslanti T, Thottakkara P, Efron PA, Feezor R, Hobson C (2015). Mortality and cost of acute and chronic kidney disease after vascular surgery. Ann Vasc Surg.

[41] Hobson CE, Ozrazgat-Baslanti T, Kuxhausen A, Thottakkara P, Efron PA, Moore FA (2015). Cost and mortality associated with postoperative acute kidney injury. Ann Surg.

[42] S & I Framework. http://wiki.siframework.org/. Accessed 10/02/2015.

[43] Patient Reported Outcomes Measurement Information System. http://www.nihpromis.org/Default - 3. Accessed 10/02/2015.

[44] Agency for Healthcare Research and Quality. Common Formats. http://www.pso.ahrq.gov/common. Accessed 10/02/2015.

[45] Drawz PE, Archdeacon P, McDonald CJ, Powe NR, Smith KA, Norton J (2015). CKD as a model for improving chronic disease care through electronic health records. Clin J Am Soc Nephrol.

[46] National Health Service. Risk to patient safety of not using the NHS Number as the national identifier for all patients. 2009. http://www.nrls.npsa.nhs.uk/resources/?entryid45=61913. Accessed 10/02/2015.

[47] Australian Goverment Department of Human Services. Healthcare Identifiers Service. http://www.humanservices.gov.au/customer/services/medicare/healthcare-identifiers-service. Accessed 01/06/2016.

[48] The Office of the National Coordinator for Health Information Technology. Federal Health IT Strategic Plan 2015-2020 2015. http://healthit.gov. Accessed 9/30/2015.

[49] CaliforniaHealthline. Three Major Systems Join San Diego Health Connect, Giving HIE Big Boost. 2015. http://www.californiahealthline.org/insight/2015/three-major-systems-join-san-diego-health-connect-giving-hie-big-boost. Accessed 12/27/2015.

[50] Booske BC, Athens JK, Kindig DA, Park H, Remington PL (2010). Different perspectives for assigning weights to determinants of health. county health rankings working paper.

[51] Patel V, Barker W, Siminerio E (2014). Individuals’ access and use of their online medical record nationwide. ONC Data Brief, no.20. Office of the National Coordinator for Health Information Technology.

[52] HealthIT.gov. Blue Button. https://www.healthit.gov/patients-families/your-health-data. Accessed 9/30/2015.

[53] United States Renal Data System. 2014 Annual Data Report: Atlas of End-Stage Renal Disease in the United States. National Institutes of Health, National Institute of Diabetes and Digestive and Kidney Diseases, Bethesda, MD. 2014. http://www.usrds.org/2014/view/Default.aspx. Accessed 12/27/2015.

[54] Sautenet B, Caille A, Giraudeau B, Leger J, Vourc'h P, Buchler M (2015). Deficits in information transfer between hospital-based and primary-care physicians, the case of kidney disease: a cross-sectional study. J Nephrol.

[55] Wilson FP, Shashaty M, Testani J, Aqeel I, Borovskiy Y, Ellenberg SS (2015). Automated, electronic alerts for acute kidney injury: a single-blind, parallel-group, randomised controlled trial. Lancet.

[56] Stukel TA, Fisher ES, Wennberg DE, Alter DA, Gottlieb DJ, Vermeulen MJ (2007). Analysis of observational studies in the presence of treatment selection bias: effects of invasive cardiac management on AMI survival using propensity score and instrumental variable methods. JAMA.

[57] Moscoe E, Bor J, Barnighausen T (2015). Regression discontinuity designs are underutilized in medicine, epidemiology, and public health: a review of current and best practice. J Clin Epidemiol.

[58] Stuart E, Huskamp H, Duckworth K, Simmons J, Song Z, Chernew M (2014). Using propensity scores in difference-in-differences models to estimate the effects of a policy change. Health Serv Outcome Res Methodol.

[59] Macedo E, Bouchard J, Mehta RL (2008). Renal recovery following acute kidney injury. Curr Opin Crit Care.

[60] Kellum JA (2014). How can we define recovery after acute kidney injury? Considerations from epidemiology and clinical trial design. Nephron Clin Pract.

